# Supernumerary intranasal tooth: case report and review of the literature

**DOI:** 10.1093/jscr/rjad537

**Published:** 2023-09-30

**Authors:** Alya Isam Elgamri, Omer Elfatih Haj Siddig, Afnan W M Jobran

**Affiliations:** Department of Paediatric Dentistry, Faculty of Dentistry, University of Khartoum, Khartoum, Sudan; Department of Oral and Maxillofacial Surgery, Faculty of Dentistry, Alzaiem Alazhari University, Khartoum, Sudan; Al-Quds University, East Jerusalem, Palestine

**Keywords:** intranasal tooth, nasal tooth, supernumerary

## Abstract

The supernumerary intranasal teeth are unusual phenomena. They may be asymptomatic or present with different signs and symptoms such as epistaxis, infection, and nasal obstruction. We report a case of a supernumerary intranasal tooth that erupted more than 2 years ago and was treated with surgical removal under local analgesia.

## Introduction

Supernumerary teeth are teeth that are added to the normal number of teeth and can be in both primary and permanent dentition; their prevalence ranges from 0.1%–1% of the population [1]. It has a small predilection of occurrence in the anterior maxilla and affects 11% of females and 15% of males between 1 and 12 years of age, with the first and third years being affected the most [2]. A supernumerary tooth may erupt intraorally in a variety of positions, such as mesiodens, paramolars, and distomolars [3]. However, the ectopic eruption of supernumerary teeth at sites other than the oral cavity is uncommon. There are case reports of ectopic eruptions of the supernumerary at the maxillary sinus [4], chin [5], and nasal cavity [6]. Some of the proposed theories tried to untie the obscurity regarding the intranasal eruption of teeth in general, including the developmental origin theory, primates with three incisors theory, and neural crest migration defect theory [7]. The ectopically erupted tooth may be deciduous, permanent, or supernumerary [8]. The ectopic eruption might be associated with other developmental anomalies such as cleft palate [9].

The difficulty in diagnosing intranasal teeth is that they have no clinical symptoms, and if symptoms are present, they can be ambiguous [10]. Although ectopic eruption is mostly an incidental finding on routine clinical or radiological examination [10]. Supernumerary teeth can cause nasal obstruction, nasolacrimal duct obstruction, epistaxis, abscess, headache, foul smell, facial pain, and crusting of the nasal mucosa [11]. For radiographic examination, periapical and orthopantomography views are the most preferred view; however, recently computed tomography scan is applied in some of the cases [6]. Radiographs of the intranasal teeth often reveal a radiopaque mass that resembles a tooth [12]. The definitive treatment of the intranasal tooth in most cases is surgical removal [10].

## Case report

**Figure 1:**
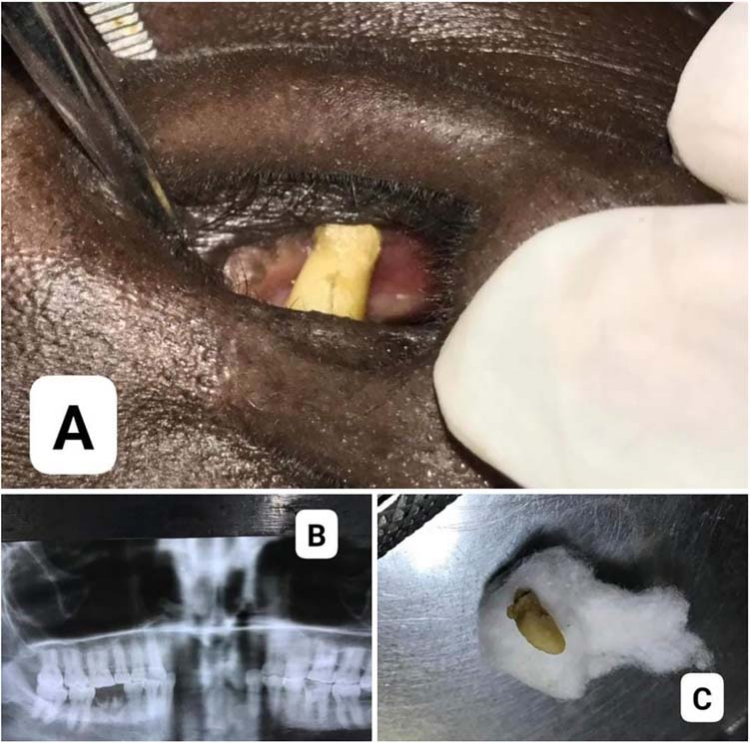
(A) Tooth-like structure protruding from the nasal septum; (B) radiographic appearance of a tooth-like structure in the right nasal cavity; (C) the supernumerary tooth after surgical removal.

A 46-year-old woman presented to our dental clinic with a 2-year history of a right nasal obstruction. She was medically fit and had a history of extraction of her upper and lower anterior teeth when she was a teenager as part of a traditional practice performed in South Sudan. Clinical examination revealed normal oral structure. However, there was a white hard mass (tooth-like material) protruding from the nasal septum toward the right nostril (Fig. 1A). Radiographic examination using orthopantomography revealed a radiopaque mass in the right nasal cavity (Fig. 1B). The treatment plan was surgical removal, determined by an oral and maxillofacial surgeon. The tooth-like structure was removed (Fig. 1C) through the right nostril under local anesthesia. An infraorbital nerve block on the right side was administered with 2% lidocaine with 1:80 000 adrenaline. The tooth-like structure was removed using curved Kelly forceps with the aid of a nasal speculum. The extracted tooth-like structures underwent histological undecalcified processing for light microscopic examination. This revealed a normal tooth histology, confirming the diagnosis of the supernumerary intranasal tooth. On follow-up examination 3 weeks later, the patient was symptom-free.

## Discussion

Supernumerary teeth are common developmental anomalies. The etiology of supernumerary teeth may be due to the development of an extra tooth bed that arises from the dental lamina, or it could be due to the division of the permanent bud [13]. Eruption of supernumerary teeth to sites other than the oral cavity is rare. Eruption of supernumerary teeth intranasally has been reported in the literature [2]. Nastri and Smith [14] reported a case of a supernumerary tooth presenting intranasally and discussed the possible diagnoses for calcified intranasal masses. Lee [15] reported a rare occurrence of an intranasal tooth associated with septal perforation. Clinically, intranasal teeth can present protruding white tooth-like materials that may be surrounded by debris. On radiographs, intranasal teeth present radiopaque structures that occupy the nasal fossa with the same attenuation as that of the oral teeth [12].

The differential diagnosis of intranasal teeth should include several conditions, such as fungal infection, osteoma, enchondroma, and malignant lesions such as osteosarcoma [12]. Although the intranasal tooth might be asymptomatic, early removal is recommended to decrease the anticipated morbidity, such as secondary infection [10]. Previous studies have recommended the removal of intranasal teeth even in symptom-free cases [10]. However, Arunkumar *et al*. [16] reported a case of a nasal tooth in which the patient refused extraction because he did not have any complaints, except for a foreign body sensation in the nose. The tooth was observed only kept under observation. Surgical removal of nasal teeth is usually a minor surgical procedure performed under local anesthesia. However, if the tooth presents with a bony socket or is accompanied by a pathological lesion such as a granuloma or cyst, the procedure may be performed under general anesthesia.

## Conclusion

A supernumerary nasal tooth has been reported in a 46-year-old woman. The tooth had erupted 2 years ago and caused nasal obstruction. Clinical and radiographic examinations were highly characteristic of supernumerary intranasal teeth. Surgical removal of the tumor was successfully performed.

## Conflict of interest statement

The authors certify that they have no affiliations with or involvement in any organization or entity with any financial or non-financial interest in the subject matter or materials discussed in this manuscript.

## Funding

None declared.
